# Effect of Local Vibration and Thoracolumbar Orthosis on Thoracic Kyphosis Angle and Trunk Flexor and Extensor Muscles in Older Adults With Hyperkyphosis: A Randomized Control Trial

**DOI:** 10.1002/hsr2.71051

**Published:** 2025-07-16

**Authors:** Fatemeh Keshavarzi, Mokhtar Arazpour, Iraj Abdollahi, Akbar Biglarian, Saeed Behzadipour

**Affiliations:** ^1^ Student Research Committee University of Social Welfare and Rehabilitation Sciences Tehran Iran; ^2^ Orthotics and Prosthetics Department University of Social Welfare and Rehabilitation Sciences Tehran Iran; ^3^ Iranian Research Center on Aging University of Social Welfare and Rehabilitation Sciences Tehran Iran; ^4^ Physical Therapy University of Social Welfare and Rehabilitation Sciences Tehran Iran; ^5^ Department of Biostatistics and Epidemiology, Social Departments of Health Research Institute University of Social Welfare and Rehabilitation Sciences Tehran Iran; ^6^ Mechanical Engineering Department Sharif University of Technology Tehran Iran; ^7^ Djawad Movafaghian Research Center in Rehab Technologies Sharif University of Technology Tehran Iran

**Keywords:** abdominal muscles, back extensor muscles, local vibration, muscle strength, spinal orthoses, thoracic kyphosis

## Abstract

**Background and Aims:**

Adding a local vibration system to a semirigid thoracolumbar orthosis may improve the effectiveness of the orthosis on muscle function in seniors with age‐related hyperkyphosis.

**Methods:**

This study was a parallel two‐arm randomized controlled trial. Eighteen seniors with age‐related hyperkyphosis were randomized into the conventional semi‐rigid thoracolumbar orthosis group (control group) or the conventional semi‐rigid thoracolumbar orthosis plus local vibration (intervention group); from June 10, 2023 to December 21, 2023. The outcomes included the thoracic kyphosis angle (TKA), parameters of isometric, isotonic, and isokinetic trunk muscle function; the SF‐36 questionnaire; the 2‐min walk test (2 MW); the 10‐m walk test (10 MW); and the timed up and go test (TUG). Dependent variables were compared between groups across three time points using mixed‐model ANOVA. One‐way repeated‐measures ANOVA was applied for significant time interactions.

**Results:**

After 6 weeks, the TKA significantly improved (*p* = 0.001) in both groups and showed more reduction in the intervention group (*p* = 0.001). The average torque of the trunk isometric extensor (*p* = 0.005) and flexor (*p* = 0.027) significantly improved in both groups. The average power of the isotonic extensors (*p* = 0.018) and flexors (*p* = 0.024) improved significantly. Isokinetic work for extensors (*p* = 0.030), flexors (*p* = 0.021) and average power (*p* = 0.017) increased.

**Conclusion:**

TKA significantly improved after 6 weeks of intervention. This improvement was significantly more in the intervention group. Adding local vibration may improve the isotonic and isokinetic torques and time parameters but not the isometric parameters of the trunk muscles.

## 1 Introduction

The normal thoracic kyphosis angle (TKA) can increase with age‐related changes in the spine. After the age of 60, approximately 40%–44% of older adults experience the increased TKA [1, 2]. A TKA, more than 50° in older adults, is considered age‐related hyperkyphosis (ARH) [3]. The prevalence of ARH is approximately 28% in women and 14% in men [4]. ARH is a progressive disorder that can increase by 3° each year [5]. This deformity can increase the risk of depression, physical function loss, balance disorders, falling, and mortality in older adults [2]. Focusing on the prevention and treatment of ARH is vital for protecting seniors' independence during their lifespan.

Among the main risk factors for ARH, the strength of the back extensor muscles has the greatest potential to limit the hyperkyphosis in older adults [2]. Back extensor muscle strengthening is the main aim of conservative interventions, including orthotics [6]. Semirigid thoracolumbar orthoses significantly affects TKA.

The semi‐rigid thoracolumbar orthoses (SRTLO), such as the Spinomed (Medi‐Bayreuth, Bayreuth, Germany), feature a leaf spring paraspinal bar. The spring bar or (bars) can align with the spin curves by the orthotist. This alignment will remain consistent throughout the user's daily activities, enabling gross trunk movements. The SRTLO function by utilizing the energy‐storing properties of leaf springs, which store energy and return it to the wearer during flexion and extension [7]. SRTLO can reduce thoracic kyphosis angle and improve the isometric strength of the back extensor muscles [8]. It also enhances the isometric strength of the trunk flexor muscles more than extensors [9]. However, in a systematic review and meta‐analysis examining the effectiveness of various conservative interventions for hyperkyphosis, the effect size of orthotic interventions was approximately half that of supervised training [10]. Another study compared a semirigid thoracolumbar orthosis with supervised training and confirmed this report [11]. Therefore, adding a supplementary intervention to promote the orthosis effect on trunk extensor muscles may improve the effect size of orthotic interventions. While many studies have investigated the function of orthotics on trunk extensor muscles [4], fewer have focused on flexor functions [8, 9] and reported a considerable increase in flexor function. Changes in hyperkyphosis angle of older adults can also affect the gait parameter [12]. Based on findings from earlier studies, it seems that there are still aspects concerning the effectiveness of SRTLO on trunk flexor and extensor muscles that would benefit from further investigation.

The differences in the morphology, position, and size of the trunk muscles require the use of various modalities for each muscle group [8, 9]. Local vibration as a therapeutic modality [13] is compatible with semirigid thoracolumbar orthosis without changing the structure of the orthosis [14]. The aim of using local vibration in this study is muscle strength improvement [15]. Previous studies on orthotic interventions reported isometric contraction of the trunk flexor and extensor muscles and improvements in endurance and proprioception [6]. However, we may need to evaluate the dynamic function of muscles to understand more aspects of the consequences of orthotic intervention on trunk movements.

There is a correlation between trunk extensor and flexor muscles' isometric, isotonic, and isokinetic power of the spinal muscles [16]. While there is a correlation between spinal fractures, trunk extensor muscle function, and TKA; trunk flexor strength is related to trunk motion control and falling [17]. This relationship confirms the effect of both muscle groups on trunk position control and the importance of both groups' evaluation to understand the effect of intervention on TKA. So, to assess the impact of incorporating a local vibration system to enhance TKA and back extensor muscle strength on the SRTLO, it is essential to evaluate both the trunk extensor and flexor muscle groups. This evaluation should include isometric and dynamic tests of trunk muscles, gait parameters, and physical function, comparing the results with those obtained from conventional SRTLOs.

This approach will provide a clearer understanding of the effects of this intervention. Therefore, this study aimed to evaluate the effects of SRTLO (control group) and SRTLO + local vibration (intervention group) on trunk extensor and flexor muscle group isometric, isotonic, and isokinetic function; TKA; Ten‐meter‐walk test, Two‐minute‐walk test, TUG, and SF‐36 questionnaires in seniors with ARH.

## 2 Methods

### 2.1 Ethics Statement

**Figure 1:**
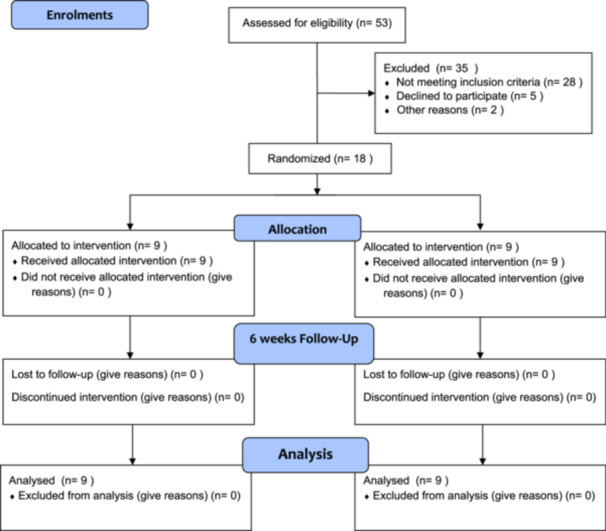
Participants recruitment flow chart.

This study was a randomized controlled clinical trial. The project protocol received approval from the ethics committee of the University of Social Welfare and Rehabilitation Sciences, with an approval code (IR.USWR.REC.1401.217) in January 2023. The protocol for this study was subsequently approved by the Iranian Registry of Clinical Trials on February 14, 2023, with the registration reference code IRCT20190811044505N2. The IRCT is a member of the WHO registry network, which emphasizes ethical conduct in clinical research. The participants were invited via flyers to workshops about ARH conducted for active groups of seniors in northern Tehran city. All evaluations were conducted in the Orthotics and Prosthetics department and cumulative Lab of the University of Social Welfare and Rehabilitation Sciences. The participant recruitment started on June 10, 2023, and ended on December 21, 2023 (Figure 1). This study was reported following the CONSORT checklist published in 2010 [18].

### 2.2 Sample Size

The number of participants calculated on the basis of TKA changes in a study with the same orthosis [19]. The number of participants in each group, calculated through the G‐power software (version 3.1, Heinrich‐Heine‐Universität Düsseldorf, Düsseldorf, Germany), includes a 95% confidence interval with an alpha error of 0.05, an effect size of 13.3, and 80% power. Each group consisted of nine participants.

#### 2.2.1 Eligibility Criteria and Recruitment

Participants included in the study based on inclusion and exclusion criteria as follows:

Inclusion criteria:

Age: participants over 60 years.
Surgical history: had undergone total knee arthroplasty (TKA) of more than 52° [4].
Body mass index (BMI): between 25 and 33.
Mobility: the ability to independently walk without assistive devices while wearing an orthosis [8].

Exclusion criteria:

Osteoporosis: individuals with a *T* score < −2.5.
Recent fractures: those with osteoporotic vertebral fractures in the past 6 months.
Hyperkyphosis: participants with hyperkyphosis due to conditions such as hemivertebra, Scheuermann disease, scoliosis, vertebral canal stenosis, tumors and infections [19].
Muscle weakness: individuals on medications for muscle weakness.
Diabetes: participants with diabetic conditions that could affect muscle function [20].
Spinal issues: those suffering from spinal degenerative diseases or experiencing nerve pain in the back and lower limbs [21].

### 2.3 Randomization, Blinding, and Treatment Allocation

Eligible participants were divided into two groups via block randomization while they were blinded to the random assignment.

### 2.4 Intervention

**Figure 2:**
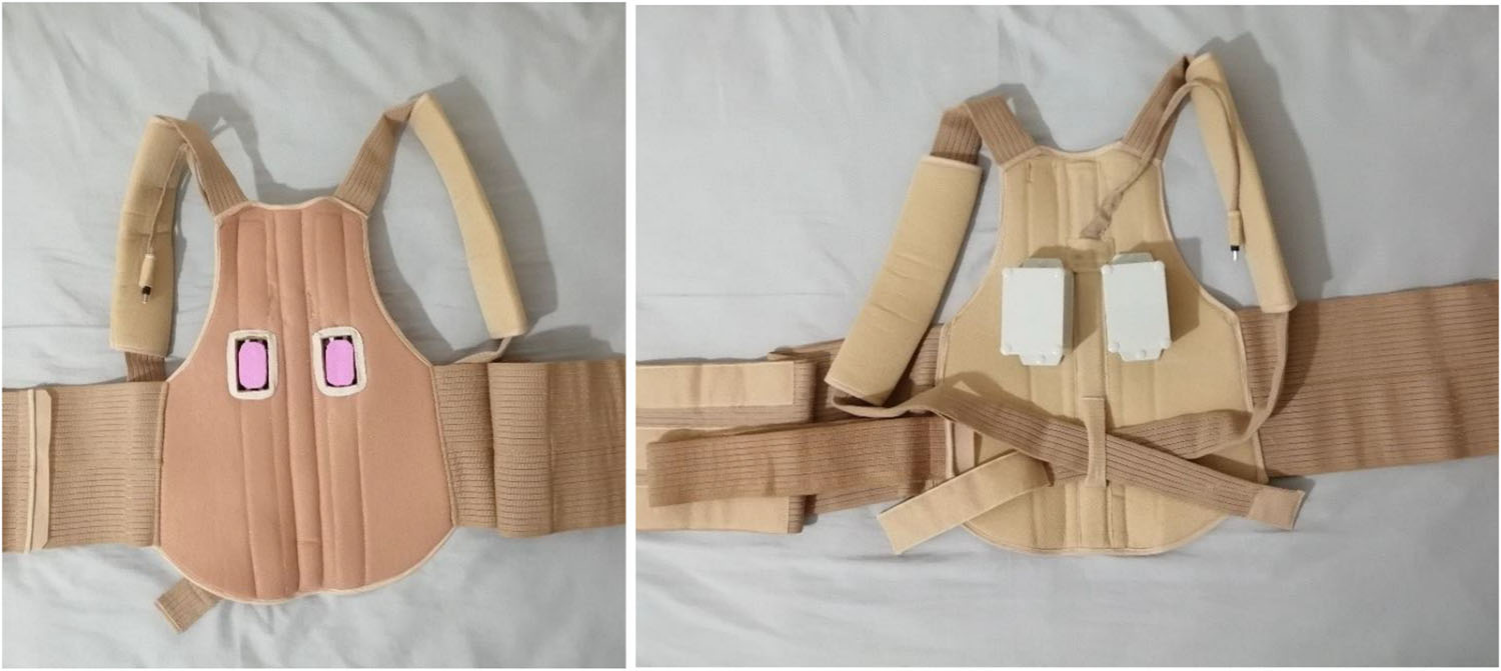
Orthosis for the intervention group. The left side displays the back view, while the right side showcases the inside view.

**Figure 3:**
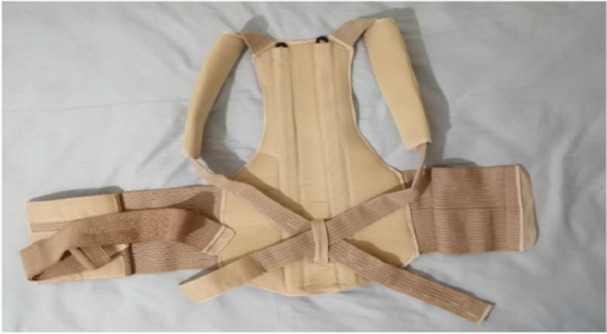
Orthosis for the control group back view.

Both groups received custom‐made SRTLO tailored to each participant's size. Compared with the no‐orthosis group, the SRTLO was previously evaluated in an RCT [19]. In this study, we equipped the SRTLO with a customized local vibration system (Figures 2 and 3). Control group received common SRTLO. Intervention group received SRTLO plus a programmed local vibration system. The duration of orthosis started at 10 min and increased from 2 weeks to 1‒2 h daily for 6 weeks [19]. The wearing time could be separated on the basis of the participants' decision [19]. Local vibration was used every other day.

**Figure 4:**
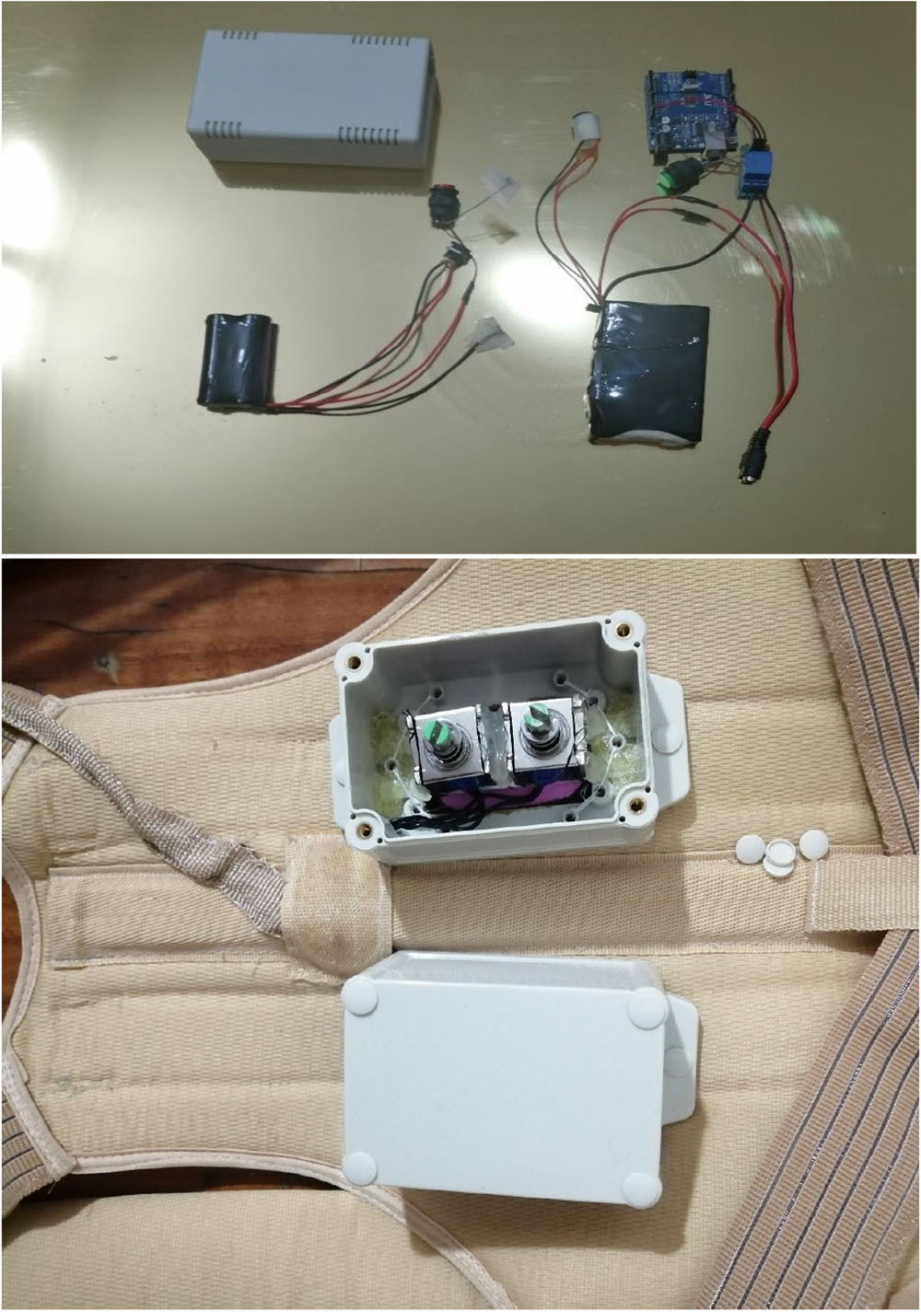
The details of vibration system. Upper image shows the box of batteries and Arduino. The lower image shows the inside of boxes positioned on the orthosis for vibration.

The vibration system is controlled by an Arduino board, which programs the vibration on the basis of a specific protocol that includes action times and rest durations. The local vibration system had four vibration units. Each unit had 3–6 mm of on‐body and 1 cm of free displacement. The vibration frequency was 8 Hz, with 20 N of vertical force, working with 300 mA current and 12 V voltage. The area of the vibration probe was 12.56 cm^2^. Vibration units were located on paraspinal muscles between the 6th and 12th thoracic vertebrae. The vibration duration was 10 s, with a 5‐min rest interval and 12 repetitions (Figure 4). There have been no previous studies on the effects of local vibration on trunk extensor muscle function or standard protocols for local vibration [22]. Therefore, we considered the caution of using local vibration for a long duration [23], and this protocol was based on studies focused on muscle function with the near thickness of the erector spine [24].

### 2.5 Outcome Measures

The primary outcomes were TKA, the 2‐min walk test and trunk flexor and extensor muscle group function. The secondary outcomes were the TUG test, the SF‐36 questionnaire and the ten‐meter walk test.

### 2.6 Evaluation Procedure

Participants visited the university's O and P department. Evaluations began with the TKA while a professional orthotist adjusted the semirigid spinal bars. The participants wore the orthosis for 30 min and then completed a checklist and usage instructions before a ten‐meter walk test. To ensure that senior participants comprehended the information presented and utilized the orthosis effectively, a checklist was employed. This checklist addressed questions related to the application of the orthosis following training. Upon mastering the correct usage of the orthotic device, participants were provided with a manual to monitor their daily wearing duration. Furthermore, this manual contained the contact information of the first author, enabling participants to seek assistance or report any discomfort or adverse effects experienced during or after the study. Isokinetic trunk settings were recorded for future sessions. The participants had 10‐min rest intervals during which they filled out the SF‐36 questionnaire. After the last isokinetic test, they rested for another 10 min before the 2‐min walk test outdoors. Outcomes were assessed before the intervention and again 3 and 6 weeks later. The SF‐36 score was evaluated before and 6 weeks after the intervention.

### 2.7 Isokinetic Dynamometer

**Figure 5:**
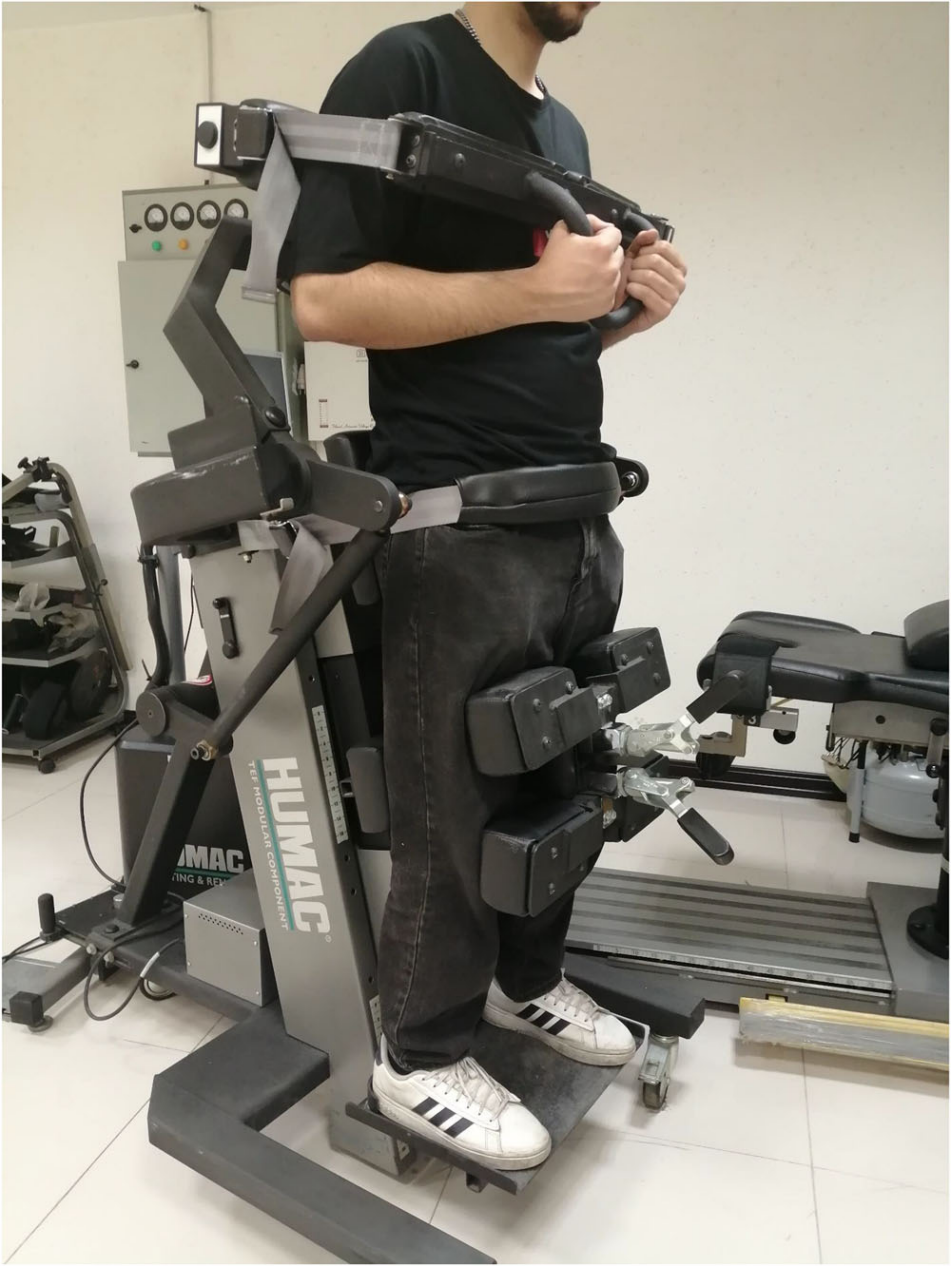
The isokinetic dynamometer (the image has no scale editing).

Trunk muscle function was evaluated with isometric, isotonic, and isokinetic tests with the trunk muscle evaluation setting of the HUMAC‐Norm isokinetic dynamometer system (CSMi, Stoughton, MA, Software HUMAC 2014, v.12.001.0005: NORM) in the standing (orthostatic) position (Figure 5). The reliability of this system for evaluating trunk muscle strength in the standing position is 0.98 [25]. The isokinetic was calibrated for each evaluation in 30° of range of motion for trunk flexion and extension with a defined 5° extension and a defined 30° flexion [26] that was chosen on the basis of the trunk kinematics of older adults [27]. The data were processed with gravity correction [28] with a speed of 60°/s [29, 30]. The positions of the anterior superior iliac spine and the seventh thoracic vertebra were adjusted for each participant according to the dynamometer's mechanical axis. Body weight, height, and adjustments for leg height and pelvic, thoracic, and knee pad positions were recorded for each participant's evaluations [31]. Once all stabilizers were securely in place, the examiner checked the participant's comfort before starting any tests.

### 2.8 Trunk Extensor and Flexor Isometric Test Protocol

The protocol consisted of two sections: a submaximal warm‐up and a test for both trunk extension and flexion. The warm‐up and test protocols were similar [32]. The warm‐up was followed by the test for 60 s. Each test included three trials lasting for 1 min with a 30‐s rest between each. No torque threshold was set, and the dynamometer was positioned at 15° of flexion. The participants received no visual feedback and were motivated by the phrase “Go as fast and as hard as possible.” After the six extension trials (three warm‐ups and three main trials), there was a 1‐min rest before the flexion tests, which followed the same protocol. After all trials were completed, a 10‐min rest was given. The HUMAC software recorded variables such as peak torque, average torque, peak torque slope, time to half peak torque, and time to peak torque.

### 2.9 Trunk Extensor and Flexor Isotonic Eccentric/Concentric Test Protocol

The trunk extensor and flexor isotonic test consists of two protocols: a submaximal familiarization trial and an actual test. Tests are conducted consecutively in the eccentric and concentric modes, with a maximum of 1 min of rest between tests and 30 s between trials. The protocol involves adjusting the trunk's range of motion and using a level 1 cushion, with an evaluation torque of 10. After the participants' positions in the HUMAC dynamometer were adjusted, the first two protocols involved concentric mode familiarization and testing, starting with three consecutive trunk flexions and extensions from 30° of flexion. Following a minute of rest, the actual test was conducted. After another minute of rest, familiarization and testing in eccentric mode began from a 5° extension. During the isotonic tests, the participants received no visual feedback and executed each movement upon the examiner's request. The variables reported by HUMAC software include torque and position parameters (peak power, work per repetition, average power per repetition, and joint angle at peak power), as well as time parameters (time to peak power, peak power decay time, reciprocal delay, and delay time).

### 2.10 Trunk Extensor and Flexor Isokinetic Concentric Test Protocol

The trunk extensor and flexor isokinetic test protocol involves two concentric protocols, including submaximal familiarization and the main test. We obtained evidence for the equality of concentric and eccentric mode evaluation results [33]. Therefore, we select the concentric mode to minimize the evaluation time. The intra‐rater (ICC = 0.89–0.95) and inter‐rater (ICC = 0.95–0.98) reliability of the isokinetic trunk flexor and extensor muscle group evaluations in the standing position were reported previously [34]. Familiarization was initiated with 30° trunk flexion, comprising three trials of consecutive flexions and extensions at 60° per second [29, 35]. There were no maximum range limits set. The participants had a 30‐s rest between trials and a 1‐min break before the main test, which followed the same protocol as familiarization. No visual feedback was provided during the tests. The variables reported by HUMAC software included peak torque, work per repetition, average power, joint angle at peak power, time to peak power, peak power decay time, reciprocal delay, and delay time. Previous reliability analysis for this protocol indicated a good intraclass correlation coefficient (0.89 for trunk flexion and 0.86 for trunk extension).

### 2.11 Thoracic Kyphosis Angle

TKA was evaluated via photogrammetry, which has high test‐retest reliability (ICC = 0.97; SEM = 1.67; MDC = 4.62) [36]. This technique is valid for assessing spine curvature in adults and has been safely applied in older adults [37]. In this study, the participants stood on a marker, whereas a calibrated camera (Canon 8 Mpixel MV150i) [38] took three digital pictures of the markers placed on the seventh cervical and 12th thoracic spinous processes [39]. The images were analyzed via AutoCAD to calculate the angle from the markers, with the average of the three angles recorded as the TKA.

### 2.12 Ten‐Meter Walk Test

The test has excellent test‐retest reliability (ICC = 0.93–0.91) [40] and interrater reliability (ICC = 0.95–0.97) [41] in healthy older adults, making it a valid measure of walking speed. We recorded the walking time for each participant on an even walkway via a stopwatch [42]. The participants walked from one marked point to another at their self‐paced speed while wearing regular footwear [43], with the examiner following closely to minimize pacing effects [44]. This test was conducted indoors.

### 2.13 Two‐Minute Walk Test

This test has excellent interrater reliability (ICC = 0.95–0.97) (37) in healthy older adults and has strong validity with other functional tests (*r* ≥ 0.84) [44]. The participants walked at their self‐paced speed from a clear marker in a straight, even outdoor walkway and wore regular footwear while the examiner followed to minimize pacing effects. At 2 min, the examiner signals participants to stop and measures the distance from the marker [44].

### 2.14 The 36‐Item Short Form Survey (SF‐36)

This test was developed for self‐reported health assessments in the general population and includes eight domains: physical activities, social activities, role activities, bodily pain, general mental health, emotional problems, vitality, and general health perceptions [45]. The Persian version of the SF‐36 has shown acceptable reliability (Cronbach's alpha = 0.70–0.85) and validity (test‒retest coefficients = 0.43–0.79) [46, 47]. It is valid for older adults under 75 years of age [48], with an 82% response rate [49]. The participants completed the questionnaire independently in private rooms, with the examiner available for assistance. The results were recorded on the basis of SF‐36 scoring instructions.

### 2.15 Pain

Pain was assessed via a reliable visual analog scale (ICC = 0.97; CI = 0.96–0.98) [50] to ensure that the participants experienced no significant pain and that the orthosis had no adverse effects.

## 3 Data Analysis (Statistics) Section

Statistical analyses were conducted using SPSS version 22. Baseline characteristics were summarized using medians and interquartile ranges (IQR) for continuous variables and counts/proportions for categorical variables. For randomized trials, baseline comparisons between groups were reported descriptively without hypothesis testing, as any observed differences are expected due to chance.

The primary analysis used a two‐way mixed ANOVA to evaluate group × time interactions across three time points, with Bonferroni correction applied to post hoc tests for significant interactions. This correction was prespecified to address multiplicity. Effect sizes (Cohen's *d*) and 95% confidence intervals (CIs) were calculated for mean differences to quantify magnitude. All tests were two‐sided with *α* = 0.05. All analyses adhered to a prespecified protocol, avoiding data‐driven changes to methods [51]. Figures were generated in R (v4.5.0) using the lattice package [52], ensuring clarity and truncation of unstable estimates where applicable.

## 4 Results

### 4.1 Participants Characteristics

From June 2023 to December 2023, 53 older adults were screened, and 18 (17 women, 1 man) were enrolled after providing informed consent. Groups differed in gender distribution (*χ*² test, *p*< 0.05) but not in other baseline characteristics (*t*‐tests, *p*> 0.05; Table 1).

**Table 1:** Distribution of demographic data in baseline.

Baseline demographic characteristics	Intervention group	Control group	*p* value
Number of participants	9	9	—
Gender	9 w	8 w ‐ 1 m	0.036*
Age (year)	64.11 ± 5.28	64.56 ± 6.31	0.498
Height (m)	1.60 ± 0.09	1.65 ± 0.12	0.366
Weight (kg)	67.11 ± 10.12	68.22 ± 9.16	0.597
BMI (kg/m^2^)	26.38 ± 3.30	24.93 ± 1.88	0.103
Pain (visual analog scale score)	0.56 ± 1.13	0.44 ± 1.01	0.598

*statistically significant.

### 4.2 Intervention Outcomes

All participants completed the 6‐week intervention. A two‐way mixed ANOVA revealed significant group × time interactions for several outcomes, indicating that changes over time differed between the control and intervention groups. Notably, thoracic kyphosis angle (TKA) decreased by a mean of 17° in the control group and 16° in the intervention group (*p *= 0.001). The 2‐min walk test distance also showed a significant interaction (*p *= 0.003), though the mean change was less than one unit ( < 1 unit), suggesting limited clinical impact. Muscle performance measures, including isotonic concentric extensor peak power (*p *= 0.02), flexor average power (*p *= 0.002), extensor work per repetition (*p *= 0.04), flexor work per repetition (*p *= 0.006), isokinetic concentric flexor work per repetition (*p *= 0.03), and extensor average power (*p *= 0.02), exhibited significant interactions (Table 2).

**Table 2:** Mean ± SD and dependent variables changes during 6 weeks in groups.

	Baseline evaluation		After 3 weeks		After 6 weeks		Changes after 3 weeks		Changes after 6 weeks	
	Common group	Intervention group	Common group	Intervention group	Common group	Intervention group	(95% CI)	*p* value	(95% CI)	*p* value
Isometric extensors peak torque	27.88 ± 22.45	24.22 ± 16.52	32.77 ± 12.89	43.66 ± 22.10	40.33 ± 22.28	49.77 ± 24.99	12.167	0.017*	−19.0	0.005*
Isometric flexors peak torque	46.44 ± 21.77	46.22 ± 18.51	44.66 ± 19.55	57.00 ± 17.93	52.00 ± 23.14	62.33 ± 18.52	−4.500	0.279	−10.833	0.027*
Isometric extensors average torque	19 ± 16.35	17.88 ± 10.65	24.77 ± 10.03	37.77 ± 18.61	32.11 ± 18.07	39.11 ± 18.69	−12.833	0.004*	−17.167	0.001*
Isometric flexors average torque	35.33 ± 14.02	38 ± 14.78	35.33 ± 13.64	45 ± 13.17	40.77 ± 16.93	49.22 ± 12.86	−3.500	0.255	−8.333	0.040*
Isometric extensors pick torque slope	11.28 ± 19.65	23.44 ± 37.09	29.11 ± 44.54	43.88 ± 41.52	29.33 ± 47.38	23 ± 7.92	31.66	1	42	0.722
Isometric flexors pick torque slope	15.77 ± 6.32	17.66 ± 10.60	16.33 ± 5.02	38.22 ± 53.10	19.33 ± 6.04	24.44 ± 9.86	−10.55	0.576	−5.167	0.251
Isometric extensors time to peak torque	2.26 ± 1.41	2.14 ± 1.14	3 ± 1.07	2.32 ± 1.10	3.44 ± 0.97	2.52 ± 0.77	−0.457	0.561	−0.773	0.087
Isometric flexors time to peak torque	3.75 ± 1.05	3.71 ± 1.31	3.71 ± 0.88	3.48 ± 1.24	3.32 ± 1.13	3.60 ± 0.94	0.136	1	0.270	1
Isotonic concentric extensors pick power	9.77 ± 2.11	10.44 ± 2.69	11.22 ± 2.53	13 ± 2.95	10.33 ± 1.73	15.55 ± 5.57	−2.000	0.145	−2.833	0.114
Isotonic eccentric extensors pick power	11.22 ± 1.64	12.33 ± 1.93	11.11 ± 2.66	12.55 ± 2.35	10.78 ± 2.28	11.34 ± 1.50	−0.056	1	0.722	0.627
Isotonic concentric flexors pick power	11.11 ± 3.02	9.66 ± 4.66	9.55 ± 1.66	13.89 ± 2.93	8.44 ± 4.22	10.44 ± 6.22	−1.333	0.712	0.944	1
Isotonic eccentric flexors pick power	10.00 ± 2.91	8.55 ± 3.64	9.44 ± 2.92	11.11 ± 4.91	8.55 ± 2.74	12.33 ± 4.84	−1.000	1	−1.167	1
Isotonic concentric extensors average power	9.77 ± 2.27	8.44 ± 3.00	9.11 ± 3.10	12.55 ± 4.24	8.66 ± 3.00	14.22 ± 5.47	−1.722	0.633	−2.333	0.323
Isotonic eccentric extensors average power	5.33 ± 1.32	5.55 ± 0.88	5.55 ± 0.88	4.88 ± 1.26	5.11 ± 1.61	5.33 ± 1.41	0.222	1	0.222	1
Isotonic concentric flexors average power	9.77 ± 2.99	10.77 ± 3.49	8.88 ± 1.53	14.77 ± 3.59	8.66 ± 3.35	16.55 ± 5.59	−1.556	0.412	−2.333	0.249
Isotonic eccentric flexors average power	5.55 ± 2.55	5.33 ± 1.00	6.00 ± 1.93	8.22 ± 4.40	4.77 ± 0.97	5.22 ± 0.55	−1.667	0.181	−1.222	0.800
Isotonic concentric extensors work per repetition	8.11 ± 0.60	8.11 ± 0.92	8.22 ± 0.83	9.22 ± 1.20	7.88 ± 1.16	9.66 ± 2.12	−0.611	0.423	−0.667	0.654
Isotonic eccentric extensors work per repetition	4.77 ± 1.30	4.66 ± 1.11	5.11 ± 1.16	4.44 ± 1.42	4.77 ± 1.71	4.66 ± 1.22	−0.056	1	0.00	1
Isotonic concentric flexors work per repetition	7.88 ± 1.05	8.11 ± 1.53	7.55 ± 0.52	9.66 ± 1.50	7.55 ± 1.23	10.33 ± 2.35	−0.611	0.566	−0.944	0.279
Isotonic eccentric flexors work per repetition	4.66 ± 2.23	4.55 ± 1.33	5.33 ± 1.22	5.44 ± 2.83	4.44 ± 1.58	6.22 ± 3.19	−0.778	0.678	−0.722	1
Isotonic concentric extensors time to peak power	0.44 ± 0.24	0.41 ± 0.13	0.38 ± 0.22	0.29 ± 0.03	0.35 ± 0.17	0.29 ± 0.04	0.029	0.026*	0.102	0.314
Isotonic eccentric extensors time to peak power	0.98 ± 0.21	1.03 ± 0.23	0.98 ± 0.25	0.92 ± 0.17	0.87 ± 0.13	1.02 ± 0.24	0.054	1	0.060	1
Isotonic concentric flexors time to peak power	0.40 ± 0.09	0.34 ± 0.05	0.40 ± 0.12	0.35 ± 0.08	0.41 ± 0.16	0.34 ± 0.05	−0.008	1	−0.007	1
Isotonic eccentric flexors time to peak power	0.78 ± 0.15	0.79 ± 0.18	0.89 ± 0.31	0.68 ± 0.17	0.80 ± 0.13	0.64 ± 0.27	0.001	1	0.064	0.743
Isokinetic concentric extensors peak torque	3.66 ± 4.24	4.33 ± 2.00	4.11 ± 2.61	6.44 ± 4.82	5.00 ± 5.07	7.44 ± 4.92	−1.27	0.65	−2.22	0.47
Isokinetic concentric flexors peak torque	17.77 ± 13.46	6.88 ± 6.46	13.88 ± 14.44	16.88 ± 9.99	13.11 ± 16.69	15.11 ± 7.33	−3.056	1	−1.778	1
Isokinetic concentric extensors work per repetition	0.66 ± 0.50	1.11 ± 0.33	1.00 ± 0.03	1.11 ± 0.33	1.00 ± 0.01	1.55 ± 1.01	−0.167	0.122	−0.389	0.261
Isokinetic concentric flexors work per repetition	0.37 ± 0.51	0.77 ± 0.66	0.75 ± 0.70	0.77 ± 0.66	0.50 ± 0.53	1.33 ± 1.11	−0.189	1	−0.333	0.788
Isokinetic concentric extensors average power	0.77 ± 0.83	1.55 ± 0.72	1.22 ± 0.83	1.77 ± 0.66	1.00 ± 0.01	2.66 ± 2.39	−0.333	0.778	−0.667	0.562
Isokinetic concentric flexors average power	0.50 ± 0.75	1.11 ± 1.26	0.87 ± 0.99	1.44 ± 1.01	1.00 ± 1.06	1.88 ± 2.02	−0.360	0.884	−0.632	0.628
Isokinetic concentric extensors joint angle in peak torque	12.44 ± 10.71	17.33 ± 5.47	17.44 ± 7.17	16.55 ± 5.10	17.00 ± 6.61	16.55 ± 6.91	−2.111	1	−1.889	1
Isokinetic concentric flexors joint angle in peak torque	14.00 ± 12.07	17.33 ± 8.47	16.66 ± 9.02	25.77 ± 6.41	17.44 ± 10.81	24.66 ± 6.24	−5.556	0.068	−5.389	0.022
Isokinetic concentric extensors time to peak torque	0.32 ± 0.24	0.66 ± 0.24	0.51 ± 0.15	0.38 ± 0.10	0.52 ± 0.18	0.40 ± 0.12	0.044	1	0.029	1
Isokinetic concentric flexors time to peak torque	0.56 ± 0.50	0.64 ± 0.38	0.75 ± 0.39	0.76 ± 0.36	0.90 ± 0.41	0.78 ± 0.38	−0.155	0.539	−0.238	0.068
Isokinetic concentric extensors force decay time	0.38 ± 0.37	0.57 ± 0.24	0.64 ± 0.35	0.42 ± 0.25	0.65 ± 0.26	0.46 ± 0.31	−0.059	1	−0.082	1
Isokinetic concentric flexors force decay time	0.47 ± 0.11	0.36 ± 0.40	0.54 ± 0.41	0.18 ± 0.01	0.69 ± 0.35	0.20 ± 0.06	0.043	1	0.052	1
Isokinetic concentric extensors delay time	−0.03 ± 0.14	−0.15 ± 0.052	−0.02 ± 0.26	0.04 ± 0.16	−0.08 ± 0.29	−0.01 ± 0.18	−0.103	0.251	−0.056	1
Isokinetic concentric flexors delay time	0.02 ± 0.12	−0.062 ± 0.27	−0.09 ± 0.10	0.11 ± 0.31	−0.14 ± 0.15	−0.01 ± 0.26	−0.055	1	0.036	1
Isokinetic concentric extensors reciprocal delay	0.78 ± 0.72	1.16 ± 0.79	1.05 ± 0.73	0.74 ± 0.37	1.25 ± 0.61	0.75 ± 0.44	0.075	1	−0.033	1
Isokinetic concentric flexors reciprocal delay	0.67 ± 0.54	1.04 ± 0.55	1.05 ± 0.39	0.77 ± 0.25	1.12 ± 0.38	0.79 ± 0.34	−0.054	1	−0.106	1
Thoracic kyphosis angle	66.88 ± 8.68	56.44 ± 4.75	56.09 ± 8.12	46.92 ± 7.01	49.68 ± 6.87	40.54 ± 7.63	10.159	0.00*	16.55	0.00*
SF‐36	74.56 ± 10.33	78.12 ± 8.42	–	–	74.24 ± 10.26	78.25 ± 8.33	–	–	−3.55	0.436
10‐meter walk test	19.33 ± 2.73	17.33 ± 1.41	18.55 ± 2.74	16.88 ± 1.69	18.44 ± 2.69	16.88 ± 1.69	0.611	0.068	0.274	0.667
2‐min walk test	178.38 ± 26.42	152.52 ± 4.33	177.71 ± 25.2	152.86 ± 3.8	176.91 ± 22.9	151.64 ± 3.91	0.164	1	1.173	1
Time up and go test	7 ± 1.73	7.44 ± 1.42	6.55 ± 1.66	6.55 ± 1.23	6.66 ± 2.23	7 ± 1.73	0.667	0.00*	0.389	0.525

*statistically significant.

Post hoc analyses with Bonferroni correction for TKA confirmed significant reductions from baseline to 3 weeks (*p* < 0.001), baseline to 6 weeks (*p* < 0.001), and three to 6 weeks (*p* = 0.001). Within both groups, significant improvements occurred in isometric extensor peak torque (*p* = 0.005), flexor peak torque (*p* = 0.03), extensor average torque (*p* = 0.001), flexor average torque (*p* = 0.04), isotonic concentric extensor time to peak power (*p* = 0.03), flexor angle of peak power (*p* = 0.02), and timed up and go test (TUG; *p* < 0.001). These changes did not differ significantly between groups, indicating similar improvements over time.

**Figure 6:**
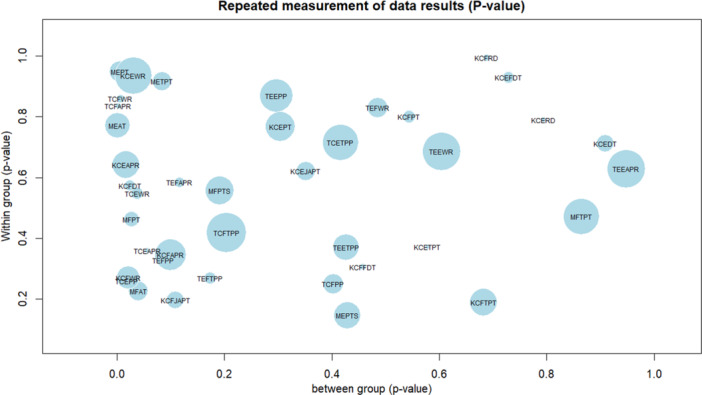
The bubble plot that illustrates interaction of time and group *p* values with the size of bubbles. MEPT = isometric extensors peak torque, MFPT = isometric flexors peak torque, MEAT = isometric extensors average torque, MFAT = isometric flexors average torque, MEPTS = isometric extensors pick torque slope, MFPS = isometric flexors pick torque slope, METPT = isometric extensors time to pick torque, MFTPT = isometric flexors time to pick torque, TCEPP = isotonic concentric extensors pick power, TEEPP = isotonic eccentric extensors pick power, TCFPP = isotonic concentric flexors pick power, TEFPP = isotonic eccentric flexors pick power, TCEAPR = isotonic concentric extensors average power per repetition, TEEAPR = isotonic eccentric extensors average power per repetition, TCFAPR = isotonic concentric flexors average power per repetition, TEFAPR = isotonic eccentric flexors average power per repetition, TCEWPR = Isotonic concentric extensors work per repetition, TEEWR = isotonic eccentric extensors work per repetition, TCFWR = isotonic concentric flexors work per repetition, TEFWR = isotonic eccentric flexors work per repetition, TCETPP = isotonic concentric extensors time to peak power, TEETPP = isotonic eccentric extensors time to peak power, TCFTPP = isotonic concentric flexors time to peak power, TEFTPP = isotonic eccentric flexors time to peak power, KCEPT = isokinetic concentric extensors peak torque, KCFPT = isokinetic concentric flexors peak torque, KCEWR = isokinetic concentric extensors work per repetition, ICFWR = isokinetic concentric flexors work per repetition, ICEAPR = isokinetic concentric extensors average power per repetition, ICFAPR = isokinetic concentric flexors average power per repetition, ICEJAPT = isokinetic concentric extensors joint angle in peak torque, ICFJAPT = isokinetic concentric flexors joint angle in peak torque, ICETPT = isokinetic concentric extensors time to peak torque, ICFTPT = isokinetic concentric flexors time to peak torque, ICEFDT = isokinetic concentric extensors force decay time, ICFFDT = isokinetic concentric flexors force decay time, ICEDT = isokinetic concentric extensors delay time, ICFDT = isokinetic concentric flexors delay time, ICERD = isokinetic concentric extensors reciprocal delay, ICFRD = isokinetic concentric flexors reciprocal delay.

**Figure 7:**
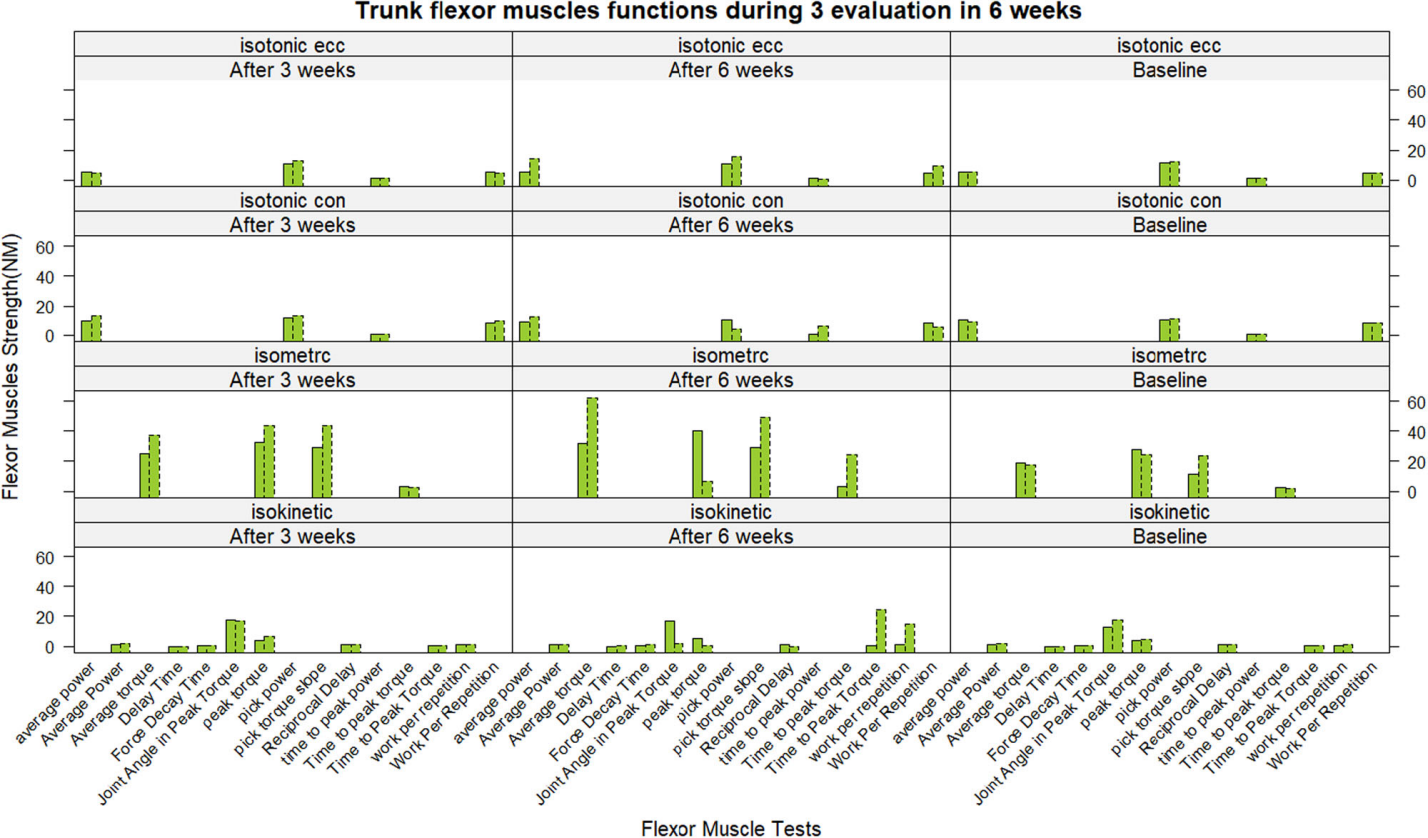
Mean changes of flexor muscles tests during the intervention. Bars with a dot edge line show the intervention group. The bars with intact edge lines represent the control group.

**Figure 8:**
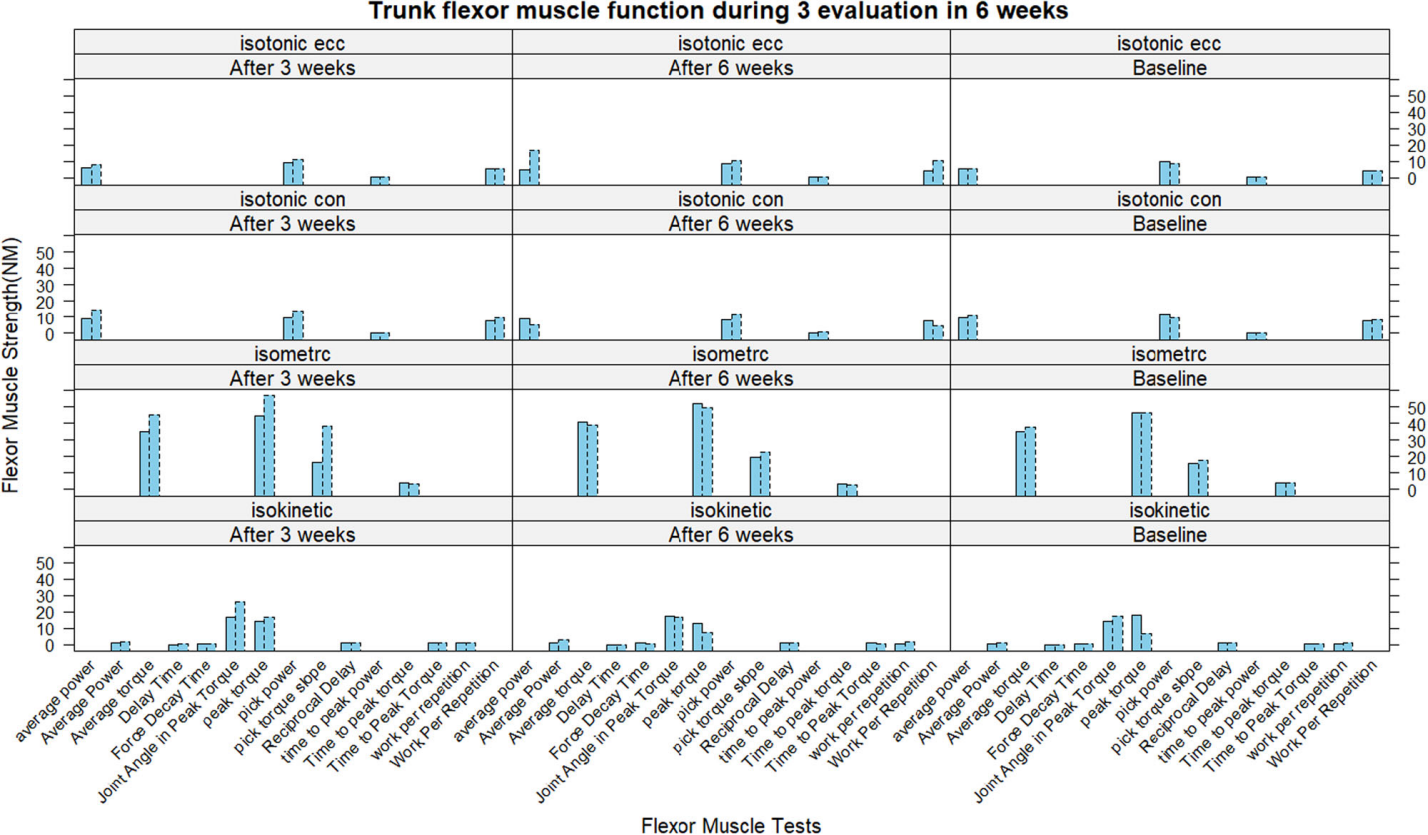
Mean changes of extensor muscles tests during the intervention. Bars with a dot edge line show the intervention group. The bars with intact edge lines represent the control group.

No participants reported pain or adverse effects from the orthosis. Detailed results, including mean changes and statistical comparisons, are presented in Tables 2 and 3, with visualizations in Figures 5, 6, 7, 8, generated using R software (version 4.5.0) and the lattice package [52]. The reporting of this study results was in respect to guideline for reporting of statistics for clinical research [51].

**Table 3:** ANOVA summary.

	Time		Group		Group × Time	
	*F*	*p* value	*F*	*p* value	*F*	*p* value
Isometric extensors peak torque	0.004	0.948	1.039	0.005	1.301	0.272
Isometric flexors peak torque	0.167	2.106	15.07	0.001*	0.028	0.868
Isometric extensors average torque	0.412	0.712	0.933	0.350	0.437	0.518
Isometric flexors average torque	0.643	0.224	3.944	0.066	0.246	0.627
Isometric extensors pick torque slope	0.001	0.971	12.425	0.003*	0.051	0.824
Isometric flexors pick torque slope	0.139	0.714	0.013	0.911	0.068	0.798
Isometric extensors time to pick torque	0.012	0.916	3.413	0.084	1.498	0.240
Isometric flexors time to pick torque	0.548	0.471	0.030	0.864	0.064	0.803
Isotonic concentric extensors pick power	1.368	0.260	7.100	0.018*	4.086	0.061
Isotonic eccentric extensors pick power	0.028	0.869	1.168	0.297	0.163	0.692
Isotonic concentric flexors pick power	1.426	0.251	0.743	0.402	1.361	0.262
Isotonic eccentric flexors pick power	1.023	0.328	3.370	0.086	4.751	0.046*
Isotonic concentric extensors average power per repetition	0.896	0.359	4.288	0.056	6.990	0.018*
Isotonic eccentric extensors average power per repetition	0.245	0.628	0.004	0.948	0.015	0.904
Isotonic concentric flexors average power per repetition	0.045	0.835	14.751	0.002*	6.323	0.024*
Isotonic eccentric flexors average power per repetition	0.311	0.585	2.785	0.116	3.755	0.072
Isotonic concentric extensors work per repetition	0.380	0.547	5.205	0.038*	3.181	0.095
Isotonic eccentric extensors work per repetition	0.170	0.686	0.281	0.604	0.013	0.911
Isotonic concentric flexors work per repetition	0.032	0.860	10.173	0.006*	4.992	0.041*
Isotonic eccentric flexors work per repetition	0.048	0.830	0.512	0.485	1.382	0.258
Isotonic concentric extensors time to peak power	0.138	0.715	0.699	0.416	0.065	0.803
Isotonic eccentric extensors time to peak power	0.853	0.370	0.671	0.426	0.652	0.432
Isotonic concentric flexors time to peak power	0.689	0.419	1.768	0.204	0.00	0.988
Isotonic eccentric flexors time to peak power	1.317	0.269	2.050	0.173	3.168	0.095
Isokinetic concentric extensors peak torque	0.092	0.766	1.134	0.304	0.378	0.548
Isokinetic concentric flexors peak torque	0.066	0.800	0.382	0.544	3.051	0.101
Isokinetic concentric extensors work per repetition	0.007	0.934	5.742	0.030*	0.032	0.860
Isokinetic concentric flexors work per repetition	1.322	0.270	6.712	0.021*	0.993	0.336
Isokinetic concentric extensors average power per repetition	0.225	0.642	7.244	0.017*	0.514	0.484
Isokinetic concentric flexors average power per repetition	0.953	0.346	3.123	0.099	0.253	0.623
Isokinetic concentric extensors joint angle in peak torque	0.253	0.622	0.927	0.351	1.516	0.237
Isokinetic concentric flexors joint angle in peak torque	1.821	0.197	2.905	0.109	1.859	0.193
Isokinetic concentric extensors time to peak torque	0.844	0.373	0.323	0.578	7.141	0.017*
Isokinetic concentric flexors time to peak torque	1.889	0.190	0.174	0.682	0.535	0.476
Isokinetic concentric extensors force decay time	0.008	0.930	0.125	0.729	3.451	0.083
Isokinetic concentric flexors force decay time	1.124	0.306	0.581	0.458	6.004	0.027*
Isokinetic concentric extensors delay time	0.142	0.712	0.013	0.909	1.883	0.190
Isokinetic concentric flexors delay time	0.331	0.574	6.259	0.024*	3.613	0.077
Isokinetic concentric extensors reciprocal delay	0.074	0.789	0.071	0.794	6.926	0.019
Isokinetic concentric flexors reciprocal delay	0.000	0.994	0.168	0.688	5.773	0.030*

*statistically significant.

## 5 Discussion

The study revealed that the intervention group outperformed the control group in TKA, walking speed, and muscle power. There were marked enhancements in isotonic strength and significant gains in work and power from isokinetic tests for both muscle groups that has clinical significancy based on previous reports of trunk muscle evaluations [28].

In this study, we found a mean reduction in the thoracic kyphosis angle (TKA) of 17.2° for the control group and 16° for the intervention group over 6 weeks. In a previous study, the same SMTLO orthosis achieved a 14.8° reduction over 12 weeks [19]. Our results were better than those of prior studies, which reported reductions of 7.9 degrees over 6 months [8] and 11.76° over 3 months with a semirigid thoracolumbar orthosis [53]. The improved outcomes may be due to the smaller number of participants and their lower average age. We also limited orthosis wear to 1–2 h daily, potentially enhancing effectiveness. Additionally, while previous research focused on a vibrant reminder for TKA [54] and reported an 8° reduction after 6 weeks [55], our study focused on trunk extensor muscle function. We used a massage therapy system designed for 3–6 mm of vertical displacement, with an 8 Hz amplitude and 10‐s vibrations followed by 5‐min rest periods to improve trunk extensor muscle efficacy [23].

The trunk extensor and flexor muscles were assessed via an isokinetic dynamometer for isometric, isotonic, and isokinetic contractions. After 6 weeks, the isometric extension peak torque changed by 34.65 Nm for the vibration orthosis and 16.78 Nm for the SRTLO orthosis. Previous research has shown greater MVC changes, such as 78.45 N over 12 weeks with the same orthosis [19]. Other SMTLO types resulted in 40 N for 10 weeks [56], 7.27 N for 12 weeks [57], 48 N [8], 17.3 N [57] and 189 N [8] for 24 weeks. Two studies reported MVC changes in trunk flexors of 66.6 N [9] and 94 N [8] over 24 weeks. The isometric peak torque of the trunk flexor in this study was 21.84 Nm in the vibration orthosis group and 7.53 Nm in the SRTLO orthosis group. Compared with sitting, standing is related to lower isometric trunk strength [58]. Muscle function improvement can be achieved during 4 weeks of intervention in older adults [59], and the trunk extensor muscle group force can increase significantly over 6 weeks [19]. While we could not reach statistical significance in the isometric tests, we reached it in the isotonic and isokinetic tests.

Previous studies did not assess the effects of spinal orthosis on trunk muscle contractions in ARH patients, but findings from healthy and older adults can provide context for our results. The peak power of trunk muscles is related to active control of the trunk during loading [60]. Isotonic contraction can improve muscle function more than isometric or isokinetic contraction because this contraction results in the recruitment of more muscle spindles at the point of fatigue [61]. We evaluated the impact of the SRTLO on the ARH, which involves daily trunk flexions and extensions that strengthen the abdominal muscles against critical loads. Following flexion, the back extensor muscles engage during extension. Compared with the standard SRTLO, the SRTLO with vibration improved peak power for isotonic extensors and average power for flexors, suggesting that vibration may enhance extensor muscle function over time.

Local vibration in SMTLO can enhance trunk extensor function, as indicated by the relationship between thoracolumbar alignment and extensor power [62]. A greater thoracic kyphosis angle and lower peak torque of trunk flexors and extensors are reported in older adults [63]. However, in this study, the changes in peak torque were not significant. The reduced strength of trunk muscles during standing dynamic contraction tests may influence the torque values reported in this study [64].

We assessed physical function, and only the 2‐min walk test showed a significant improvement in the vibration orthosis group compared with the common group. The evidence revealed a relationship between trunk inclination and ultrathin gait in older adults but not in those who underwent TKA [65]. While the 2‐min test did not show significance post hoc, prior studies indicated improvements in the 2‐min and 10‐m tests [66]. The TUG results were not significant in our study, although previous research reported improvements with SMTLO interventions [53]. Additionally, there were no significant changes in participants' quality of life.

Considering that greater trunk extensor strength is associated with increased lumbar lordosis [67], we can interpret the faster reduction in thoracic kyphosis as unintended resistance to the increase in lumbar lordosis throughout the study.

## 6 Limitations

The sex distribution in this study was notably uneven, with only one male participant included. The higher prevalence of Age‐Related Hyperkyphosis (ARH) among women accounts for this disparity; however, it does not warrant the extrapolation of the findings to men with hyperkyphosis. The application of local vibration should be approached with caution due to the absence of standardized protocols. It is advisable to commence with a small cohort and a brief intervention duration, to develop a rigorously evaluated protocol for local vibration within the context of orthotic intervention aimed at enhancing muscle function. Additionally, generalizing these findings necessitates a larger sample size that reflects a balanced gender distribution in future research endeavors.

## 7 Conclusion

TKA significantly improved after 6 weeks of semirigid TLO. This improvement was significantly greater in the (SR‐TLO) + local vibration group. The isometric torque of the trunk flexure and extensor muscle groups improved more in the (SR‐TLO) + local vibration group than in the conventional SR‐TLO group, but this difference was not significant. Using an SR‐TLO may beneficially affect the results of the TUG and TMW tests. Compared with the conventional group (SR‐TLO), the 6‐week (SR‐TLO) + local vibration group significantly improved the TKA, the concentric and eccentric pick power and average power of the isotonic tests for flexors and extensors, and the isotonic and isokinetic concentric work per repetition of flexors and extensors.

## Author Contributions

**Fatemeh Keshavarzi:** conceptualization, investigation, writing – original draft, resources, and project administration. **Mokhtar Arazpour:** conceptualization, investigation, writing – review and editing, supervision, and project administration. **Iraj Abdollahi:** writing – review and editing, validation, and visualization. **Akbar Biglarian:** methodology, validation, writing – review and editing, data curation, and formal analysis. **Saeed Behzadipour:** writing – review and editing, software.

## Ethics Statement

This project protocol received approval from the ethics committee of the University of Social Welfare and Rehabilitation Sciences, with the approval code (IR.USWR.REC.1401.217) on January 1, 2023. Then, the protocol for this study was approved by the Iranian Registry of Clinical Trials on February 14, 2023, with registration reference code (IRCT20190811044505N2).

## Consent

All participants provided written informed consent before enrollment in the study. This study was conducted ethically in accordance with the World Medical Association Declaration of Helsinki.

## Conflicts of Interest

The authors declare no conflicts of interest.

## Transparency Statement

The corresponding author, Mokhtar Arazpour, affirms that this manuscript is an honest, accurate, and transparent account of the study being reported; that no important aspects of the study have been omitted; and that any discrepancies from the study as planned (and, if relevant, registered) have been explained.

## Data Availability

Individual participant data will be accessible. The data supporting the findings reported in this article, following de‐identification—including text, tables, figures, and appendices—will be available. The study protocol is registered in the Iranian Randomized Clinical Trial Registration System (IRCT) along with the corresponding IRCT code. The timeframe for data availability commences 9 months after publication and concludes 12 months post‐publication for requests from other faculties seeking collaboration. For individuals interested in conducting a meta‐analysis of participant data, such data will be available upon request to the first author.
